# Sociodemographic and Disease Correlates of Body Image Distress among Patients with Systemic Sclerosis

**DOI:** 10.1371/journal.pone.0033281

**Published:** 2012-03-23

**Authors:** Lisa R. Jewett, Marie Hudson, Vanessa L. Malcarne, Murray Baron, Brett D. Thombs, Canadian Scleroderma Research Group

**Affiliations:** 1 Department of Psychiatry, McGill University, Montréal, Québec, Canada; 2 Department of Epidemiology, Biostatistics, and Occupational Health, McGill University, Montréal, Québec, Canada; 3 Department of Medicine, McGill University, Montréal, Québec, Canada; 4 School of Nursing, McGill University, Montréal, Québec, Canada; 5 Lady Davis Institute for Medical Research, Jewish General Hospital, Montréal, Québec, Canada; 6 Division of Rheumatology, Jewish General Hospital, Montréal, Québec, Canada; 7 Department of Psychiatry, Jewish General Hospital, Montréal, Québec, Canada; 8 Department of Psychology, San Diego State University, San Diego, California, United States of America; The University of Hong Kong, Hong Kong

## Abstract

**Background:**

Body image concerns are infrequently studied in systemic sclerosis (SSc), even though significant visible disfigurement is common. The objective of this study was to identify sociodemographic and disease-related correlates of dissatisfaction with appearance and social discomfort among people with SSc.

**Methods:**

SSc patients came from the 15-center Canadian Scleroderma Research Group Registry. Sociodemographic information was based on patient self-report. Disease characteristics were obtained via physician examinations. The Brief-SWAP was used to assess dissatisfaction with appearance and social discomfort. Structural equation models were conducted with MPlus to determine the relationship of dissatisfaction with appearance and social discomfort with age, sex, education, marital status, race/ethnicity, disease duration, skin involvement, telangiectasias, skin pigmentation changes, and hand contractures.

**Results:**

A total of 489 SSc patients (432 female, 57 male) were included. Extent of skin involvement was significantly associated with both dissatisfaction with appearance and social discomfort (standardized regression coefficients = 0.02, p = 0.001; 0.02, p = 0.020, respectively), as was skin involvement in the face (0.18, p = 0.016; 0.23, p = 0.006, respectively). Greater social discomfort was robustly associated with younger age (−0.017, p<0.001) and upper-body telangiectasias (0.32, p = 0.021). Dissatisfaction with appearance was associated with hand contractures (0.07, p = 0.036).

**Conclusion:**

This study found that dissatisfaction with appearance and social discomfort were associated with numerous disfiguring characteristics of SSc, in addition to age. These results underline that there are multiple factors contributing to body image distress in SSc, as well as the need to attend to both disease and social contexts in understanding the impact of disfigurement among patients.

## Introduction

Systemic sclerosis (SSc), or scleroderma, is a multi-system, chronic autoimmune connective tissue disease characterized by vascular injury, immune dysfunction, and abnormal fibrotic processes. SSc affects the skin, as well as internal organs such as the lungs, heart, and gastrointestinal tract [1]. The rate of disease onset is highest between 30–50 years of age, and 80% of people affected are female [2], [3]. Median survival time from diagnosis is approximately 11 years, with patients 3.7 times more likely to die within 10 years of diagnosis (44.9% mortality) than age, sex, and race-matched individuals without the disease (12.0% mortality) [2]. The name *scleroderma* is derived from the Greek words *scleros*, meaning hard, and *derma* meaning skin. This is because a central feature of SSc is the excessive production of collagen, which manifests itself in thickening and hardening of the skin, and commonly leads to significant visible disfigurements to various body parts, including the face, mouth, and hands [1].

Disfiguring injury or illness often results in difficulty maintaining a healthy body image and decreased comfort in social interactions, both of which can lead to anxiety and other forms of distress [4], [5]. Research among patients with physical disfigurements has highlighted that characteristics of the disfigurement, including severity, visibility, and location of appearance changes, may influence body image dissatisfaction and related problems [6]. There has been relatively little research on body image distress in SSc. Nonetheless, existing studies have shown that more severe disease manifestations, such as significant skin changes in the hands, are associated with greater body image dissatisfaction, increased depressive symptoms, and reduced overall psychosocial functioning, including appearance-related anxiety and social avoidance [7]–[9]. One recent study of 171 SSc patients examined the impact of facial changes, and reported that patients rated facial disfigurement as the most worrying aspect of the condition [10], and another study of 129 SSc patients reported that greater objective disfigurement was linked to more distress, worry, and perceptions of noticeability by patients [9].

Research from non-disfigured populations has suggested that women are less satisfied with their appearance than men [6], [11], and generally, studies of body image, broadly defined, report that women tend to have poorer body image appraisals [12]. Furthermore, in the context of social interactions, particularly for establishing intimate relationships, appearance is highly relevant. Thus, appearance concerns are sometimes more salient for unattached persons compared to those in stable marriages or intimate relationships [6]. Similarly, concerns with appearance and its importance for meeting new people and making social connections is often more pronounced earlier in life, and the broader literature on people with changes to their appearance due to illness and injury has reported that older age is generally associated with less distress related to body image [13].

Patients with SSc may experience significant emotional distress due to negative self-appraisals or dissatisfaction with their appearance, discomfort in social situations due to changes in appearance, or both. Dissatisfaction with appearance and social discomfort are related, but distinct constructs [14]. The Brief-Satisfaction with Appearance Scale (Brief-SWAP) [14] was designed to evaluate both dissatisfaction with appearance and social discomfort related to disfigurement from SSc. The Dissatisfaction with Appearance subscale assesses self-reported dissatisfaction with specific body parts commonly affected by SSc (face, hands, and arms). The Social Discomfort subscale assesses the impact of body image concerns on social interactions (e.g., comfort with strangers, feelings of unattractiveness to others) [14].

The objective of this study was to identify sociodemographic (age, sex, marital status, race/ethnicity, and education) and disease factors (skin involvement, telangiectasias, hand contractures and disfigurement, and changes in skin pigmentation) associated with (1) dissatisfaction with appearance and (2) social discomfort among patients with SSc. We hypothesized that sociodemographic variables, including age, sex, and marital status, as well as SSc disease factors, including skin involvement, telangiectasias, hand contractures, and changes in skin pigmentation would be potentially associated with dissatisfaction with appearance and social discomfort.

## Methods

### Ethics Statement

This study consisted of an analysis of data from within the Canadian Scleroderma Research Group's (CSRG) pan-Canadian Registry. Ethics approval for the Registry and this study was provided by the research ethics committee of McGill University. In addition, the research ethics committees of each of the 15 CSRG centres approved data collection for inclusion in the Registry. All patients provided written informed consent.

### Participants and Procedure

The sample consisted of patients who completed annual visits as part of the CSRG Registry between 2008, when the Brief-SWAP was first included in the Registry, and 2010. To be eligible for the Registry, patients must have a diagnosis of SSc made by a Registry rheumatologist, be ≥18 years of age, and be fluent in English or French. Registry patients undergo physician evaluations at their initial visit and at subsequent yearly visits. Patients were included in the study if they had complete data for the Brief-SWAP and all other variables included in analyses. Some patients completed the Brief-SWAP at more than one visit, but only data from the first administration were analyzed in this study.

### Measures

Dissatisfaction with appearance and social discomfort were measured by the Brief-SWAP [14]. Sociodemographic variables included in analyses were assessed by patient self-report and consisted of age, sex, marital status (married or living as married versus single, divorced, or widowed), race/ethnicity (white versus other race/ethnicity), and education (high school or less versus higher education). Disease-related variables included in analyses were measured via clinical histories and examinations by study physicians and consisted of disease duration; total body skin involvement (modified Rodnan skin score, mRss), as well as body area-specific skin involvement scores; upper-body telangiectasias; skin pigmentation changes (hyper-and/or hypo-pigmentation); and fingertip-to-palm distance to assess hand contractures. In addition, disease subtype was recorded, but it was not entered into the analyses because of the high degree of overlap with mRss. Although over 1,000 variables are collected at each CSRG visit, only the variables described here were considered for inclusion in analyses.

#### The Brief-Satisfaction with Appearance Scale (Brief-SWAP)

The 6-item Brief-SWAP [14] assesses dissatisfaction with appearance and social discomfort related to disfigurement in SSc. It was adapted from the original 14-item Satisfaction with Appearance Scale (SWAP) [15] that was designed to measure non-weight related body image dissatisfaction among burn survivors. The Brief-SWAP includes two subscales: Dissatisfaction with Appearance, reflecting dissatisfaction with the appearance of specific body parts relevant to patients with SSc (face, hands, arms), and Social Discomfort, reflecting social discomfort relative to disfigurement from SSc. Respondents to the Brief-SWAP rate the degree to which they feel each item reflects their thoughts and feelings about their appearance on a 7-point scale ranging from 0 (*strongly disagree)* to 6 (*strongly agree*). Item scores can be summed to calculate total subscale scores with higher scores indicating greater dissatisfaction or social discomfort. The range of possible scores is 0–18 for each subscale. Each subscale of the Brief-SWAP is comprised of three items. Items from the Dissatisfaction with Appearance subscale are: (1) I am satisfied with the appearance of my face, (2) I am satisfied with the appearance of my hands, and (3) I am satisfied with the appearance of my arms. Items from the Social Discomfort subscale are (1) Because of changes in my appearance caused by my scleroderma, I am uncomfortable in the presence of strangers, (2) I feel that my scleroderma is unattractive to others, and (3) I don't think people would want to touch me. In a previous study of 654 SSc patients from the CSRG Registry, the internal consistency reliability was good for both the Dissatisfaction with Appearance (α = 0.81) and Social Discomfort (α = 0.81) subscales. The Brief-SWAP total score was highly correlated with the full 14-item SWAP total score (r = 0.95), and all correlations of the Brief-SWAP total score and full SWAP total score with measures of convergent validity were substantively equal with no statistically significant differences [14]. The raw sum scores of the Dissatisfaction with Appearance subscale and Social Discomfort subscale were only moderately correlated at r = 0.46 (correlation of latent factors = 0.56) [14], thus, in the current study, subscale scores, rather than a total score, were used.

#### Disease-Related Variables

SSc disease duration was defined as the time from onset of the first non-Raynaud's disease symptoms based on clinical history established by study physicians. Limited/diffuse status was recorded and defined as per LeRoy's definition. Limited disease was defined as skin involvement distal to the elbows and knees with or without face involvement and diffuse disease as skin involvement proximal to the elbows and knees and/or involving the trunk [16]. Total body skin involvement was assessed using the mRss, a previously validated method that has been used in other SSc samples [17]–[21]. Skin involvement is an important component of disease severity in SSc [17], [18]. For each of 17 body areas, physicians rate the skin from 0 (*normal*) to 3 (*hidebound*), summing to obtain the mRss; scores can range in severity from 0 to 51. In addition, to determine the most salient body areas of potential concern, area-specific scores were derived for six body regions, including (1) the face, (2) hands/fingers, (3) arms, (4) chest/abdomen, (5) thighs, and (6) legs and feet. The area-specific scores were defined by the maximum severity score for each area (possible maximum score 0–3). For instance, the arms comprised four individual parts (i.e., right upper arm, right lower arm, left upper arm, left lower arm), and we defined the variable “arms” as the highest score from any of those parts. Telangiectasias were defined as including either macular or dot telangiectasias, or both; these involve the visible dilatation of superficial cutaneous blood vessels that collapse upon pressure and fill slowly when pressure is released, and exclude normal sun exposure-related telangiectasias. This definition is based on an adapted definition from Medsger et al. [17], as there is no uniform, validated definition of telangiectasias. Telangiectasias were coded separately for the upper body and lower body as present or absent. However, we used only upper-body telangiectasias because very few patients had lower-body, but not upper-body telangiectasias (4 of 390 with any telangiectasias in the current sample, 1.0%). Hyper- and/or hypo-pigmentation of the skin were each scored by physicians as present or absent. For analyses, we coded patients as having any pigmentation change versus none due to the high degree of overlap between occurrences of hyper- and hypo-pigmentation. In the current sample, only 23 of 119 patients with hypo-pigmentation did not have hyper-pigmentation as well (19.3%). To determine the presence of possible disfigurement because of hand contractures, fingertip-to-palm distances of both hands were measured by asking patients to make a fist, and the distance from the tip of the finger pad of the third finger to the distal palmar crease in full flexion was recorded in centimetres. This is a consistently used surrogate marker of contractures that is based on established methods [17], [18]. In this study, the greater fingertip-to-palm distance of the right and left hand was used.

### Data Analysis

To simultaneously assess correlates of dissatisfaction with appearance and social discomfort, we used the software MPlus [22] and replicated the two-factor confirmatory factor analysis model that was previously reported for the Brief-SWAP (Dissatisfaction with Appearance and Social Discomfort) [14]. Item responses for the Brief-SWAP are ordinal Likert data, so the weighted least squares estimator with a diagonal weight matrix, robust standard errors, and a mean-and variance-adjusted chi-square statistic was used with delta parameterization [22]. A chi-square goodness-of-fit test and three fit indices were used to assess model fit, including the Tucker-Lewis Index (TLI) [23]; the comparative fit index (CFI) [24]; and the root mean square error of approximation (RMSEA) [25]. Since the chi-square test is highly sensitive to sample size and can lead to the rejection of well-fitting models, practical fit indices were emphasized [26]. Guidelines proposed by Hu and Bentler [27] suggest that models with TLI and CFI close to .95 or higher and the RMSEA close to .06 or lower are representative of good fitting models. A CFI of .90 or above [28] and a RMSEA of .08 or less [29] may also be considered to represent reasonably acceptable model fit.

In an initial model, we regressed the Dissatisfaction with Appearance and Social Discomfort latent factors on *a priori* specified sociodemographic (age, sex, education, marital status, and race/ethnicity) and disease-related variables (disease duration, mRss, upper-body telangiectasias, hyper- and/or hypo-pigmentation, and fingertip-to-palm distance). We removed sociodemographic and disease-related variables from the model if they were not associated with the Dissatisfaction with Appearance or Social Discomfort subscales (p>0.25), if their inclusion worsened overall model fit, and if their removal did not substantively or significantly influence links between other sociodemographic or disease variables and the Brief-SWAP latent factors.

Due to the significant association between mRss and the Dissatisfaction with Appearance and Social Discomfort subscales, in order to determine the body areas where skin involvement was most closely linked to these body image factors, in a second model, we replaced the mRss with body area-specific scores for the (1) face, (2) hands/fingers, (3) arms, (4) chest/abdomen, (5) thighs, and (6) legs and feet. We did this by initially entering the area-specific scores individually in separate models then including the body area with the most robust association with each factor. Subsequently, we kept that body area in the model and added other areas that were significant, starting with largest associations first.

## Results

### Sample Characteristics

A total of 489 patients were included in the study. Sociodemographic and disease characteristics are presented in Table 1, as well as Pearson bivariate correlations between sociodemographic and disease variables and Brief-SWAP subscales. The mean age of the sample was 57.1 years (SD = 11.7, range = 21–84 years), 88% of patients were female, and 91% were White. The mean time since onset of the first non-Raynaud's disease symptoms was 10.4 years (SD = 8.7), and the mean mRss was 9.0 (SD = 8.7; internal consistency α = 0.94). Mean mRss was significantly higher in diffuse (mean = 19.2, SD = 9.8) compared to limited disease patients (mean = 5.9, SD = 4.2; p<0.001). The Pearson correlation between limited/diffuse status and mRss was 0.65 (p<0.001). As shown in Table 2, Pearson correlations between body area-specific scores ranged from 0.32 to 0.66. The mean Dissatisfaction with Appearance subscale score was 8.4 (SD = 5.2), and the mean Social Discomfort subscale score was 5.1 (SD = 5.1). Both subscales had good internal consistency reliability (Dissatisfaction with Appearance α = 0.82, Social Discomfort α = 0.83).

**Table 1:** Sociodemographic Variables, Disease Characteristics for SSc Patients, and Pearson Correlations with Brief-SWAP Subscales (N = 489).

Sociodemographic Variables:		Correlation with Dissatisfaction with Appearance	Correlation with Social Discomfort
Age *(mean years, SD)*	57.1 (11.7)	−0.04 (p = 0.400)	−0.20 (p<0.001)
Female sex *(n, %)*	432 (88.3)	−0.01 (p = 0.818)	0.06 (p = 0.220)
White *(n, %)*	447 (91.4)	−0.03 (p = 0.516)	−0.06 (p = 0.167)
Married *(n, %)*	344 (70.3)	−0.05 (p = 0.306)	−0.06 (p = 0.205)
Above high school education *(n, %)*	227 (46.4)	0.05 (p = 0.232)	0.01 (p = 0.839)
**Disease Characteristics:**			
Disease duration *(mean years, SD)*	10.4 (8.7)	0.001 (p = 0.977)	0.01 (p = 0.877)
Limited SSc *(n, %)*	323 (71.0)	0.16 (p = 0.001)	0.21 (p<0.001)
Modified Rodnan Skin Score (0–51) *(mean, SD)*	9.0 (8.7)	0.24 (p<0.001)	0.23 (p<0.001)
Maximum Skin Scores for Specific Body Areas:			
Face (0–3) *(mean, SD)*	0.6 (0.8)	0.18 (p<0.001)	0.21 (p<0.001)
Right/left hands and right/left fingers (0–3) *(mean, SD)*	1.7 (1.0)	0.18 (p<0.001)	0.17 (p<0.001)
Right/left upper arms and right/left forearms (0–3) *(mean, SD)*	0.5 (0.8)	0.20 (p<0.001)	0.20 (p<0.001)
Chest and abdomen (0–3) *(mean, SD)*	0.2 (0.6)	0.17 (p<0.001)	0.22 (p<0.001)
Right/left thighs (0–3) *(mean, SD)*	0.4 (1.1)	0.18 (p<0.001)	0.17 (p<0.001)
Right/left lower leg and right/left feet (0–3) *(mean, SD)*	0.6 (0.9)	0.16 (p<0.001)	0.11 (p = 0.013)
Upper-body telangiectasias	386 (78.9)	0.08 (p = 0.080)	0.11 (p = 0.020)
Hyper- and/or hypo-pigmentation	210 (42.9)	0.14 (p = 0.002)	0.18 (p<0.001)
Fingertip-to-palm distance (greater of right and left hands)*(mean cm, SD)*	1.0 (1.7)	0.18 (p<0.001)	0.16 (p = 0.001)
**Body Image:**			
Brief-SWAP (*mean*, SD)	13.5 (8.8)	------	------
Brief-SWAP Dissatisfaction with Appearance Subscale	8.4 (5.2)	------	0.59 (p<0.001)
Brief-SWAP Social Discomfort Subscale	5.1 (5.1)	0.59 (p<0.001)	------

**Table 2:** Pearson Correlations between Body Area-Specific Scores^1^.

	Face	Hands and Fingers	Arms	Chest and Abdomen	Thighs	Legs and Feet
**Face**	1.00	0.47	0.48	0.43	0.33	0.32
**Hands and Fingers**		1.00	0.49	0.36	0.35	0.35
**Arms**			1.00	0.63	0.65	0.44
**Chest and Abdomen**				1.00	0.66	0.38
**Thighs**					1.00	0.51
**Legs and Feet**						1.00

1 All correlations p<0.01.

### Correlates of Dissatisfaction with Appearance and Social Discomfort

**Figure 1:**
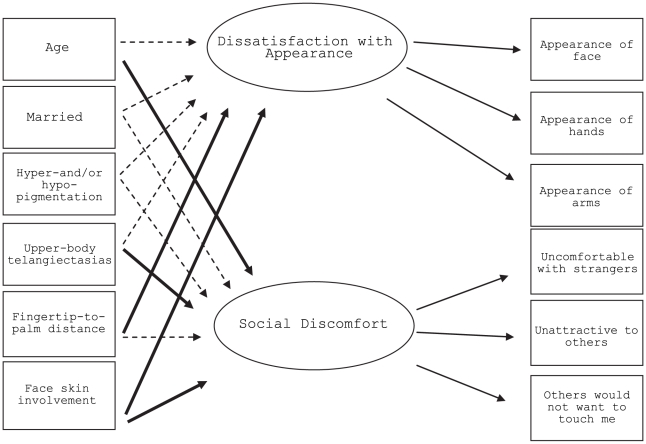
Structural equation model of relationships with sociodemographic and disease variables.

In the initial model, mRss was significantly associated with both greater Dissatisfaction with Appearance (standardized regression coefficient  = 0.02, p = 0.001) and greater Social Discomfort (0.02, p = 0.020). When we replaced the mRss in the model with body-specific areas, each was significantly associated with Dissatisfaction with Appearance and Social Discomfort latent factors (p<0.05) with face being the most robust. In the final model (see Figure 1), face skin involvement was significantly associated with both factors (Dissatisfaction with Appearance, 0.18, p = 0.016, Social Discomfort, 0.23, p = 0.006). Once scores for the face were included in the model, no other area-specific scores were statistically significant when added. Model fit was good (χ^2^(30) = 84, CFI = 0.99, TLI = 0.98, RMSEA = 0.06), and all item factor loadings were ≥0.79 for the Dissatisfaction with Appearance factor and ≥0.67 for the Social Discomfort factor. The correlation between the Dissatisfaction with Appearance and Social Discomfort factors was 0.59. The model accounted for 13% of the variance in the Social Discomfort latent factor and 6% of the variance in the Dissatisfaction with Appearance latent factor. Model parameters are shown in Table 3. Fingertip-to-palm distance, the measure of hand contractures, was significantly related to greater Dissatisfaction with Appearance (0.07, p = 0.036). Social Discomfort was significantly associated with presence of upper-body telangiectasias (0.32, p = 0.021) as well as younger age (per year) (−0.017, p<0.001). Skin pigmentation changes were not significantly associated with either factor, but there was some suggestion of an association (Dissatisfaction with Appearance, 0.15, p = 0.161; Social Discomfort, 0.22, p = 0.068). Education, race/ethnicity, and sex were not associated with either Dissatisfaction with Appearance or Social Discomfort at p<0.25, and their inclusion reduced model fit, so they were not included in the model.

**Table 3:** Brief-SWAP Structural Equation Model of Relationships with Sociodemographic and Disease Variables.

Variables	Dissatisfaction with Appearance		Social Discomfort	
	Standardized Regression Coefficients	p value	Standardized Regression Coefficients	p value
Age	−0.001	0.863	−0.017	<0.001
Married	−0.16	0.138	−0.19	0.104
Upper-body telangiectasias	0.04	0.774	0.32	0.021
Face skin involvement	0.18	0.016	0.23	0.006
Fingertip-to-palm distance	0.07	0.036	0.05	0.199
Hyper- and/or hypo-pigmentation change	0.15	0.161	0.22	0.068
**Correlation of Dissatisfaction with Appearance and Social Discomfort Factors**	0.59			

## Discussion

This study found that, in multivariate analyses, both disease factors and age were related to Dissatisfaction with Appearance and Social Discomfort among people with SSc. The findings highlighted the multifaceted nature of these two body image factors, as they were related to various disfiguring aspects of SSc, including skin involvement, upper-body telangiectasias, and disfigurement from hand contractures. Younger patients were more socially uncomfortable than older patients; however, age was not strongly related to Dissatisfaction with Appearance.

The associations of disfiguring physical changes with Dissatisfaction with Appearance and Social Discomfort are not surprising. For instance, relationships among extensive skin involvement, decreased appearance self-esteem, and heightened dissatisfaction with appearance have been documented previously in SSc [4], [30], [31] and in other types of acquired disfigurements [32]. A previous study, for instance, found that skin thickening was associated with lower appearance self-esteem in a sample of women with SSc [4]. Beyond SSc, previous research has documented social anxiety and avoidance behaviours among persons who have sustained visible disfigurement from a variety of sources to the face and other socially relevant body parts [33]. This is similarly reflected in the present study, as face skin involvement, upper-body telangiectasias, and hand contractures – salient physical changes that occur in visible and socially relevant body parts – were significantly related to either Dissatisfaction with Appearance or Social Discomfort. The current results also suggest that skin pigmentation changes may be important for both factors. It is of note that hands and fingers are visible and not easily hidden from others with clothing, but skin involvement to these areas was not associated with Dissatisfaction with Appearance or Social Discomfort in the multivariate model once face involvement was included. The hands and fingers variable was significant when face was not included, however, and the fact that it was not significant when added to the model after the face variable may be related to the relatively high degree of association between the variables.

Preoccupation with appearance and its importance for meeting new people, making social connections, and developing intimate relationships is often more pronounced earlier in life, which may account for the strong link in this study between age and Social Discomfort. We found that, although age did not play a role in individuals' ratings of Dissatisfaction with Appearance, it was an important factor in feelings of Social Discomfort. This finding is consistent with the broader literature on people with changes to their appearance due to illness and injury, which has found that older age is generally associated with less distress related to body image [13]. In the context of social interactions, particularly for establishing intimate relationships, appearance is highly relevant. Worry about a different or disfigured appearance can result in heightened focus on appearance itself, as well as its social consequences (e.g., thoughts of being disadvantaged in searching for a partner) [6]. Taken together, these findings suggest that younger patients may be at a greater risk of body image distress related to Social Discomfort based on physical appearance. However, the relatively small amount of variance explained by sociodemographic variables and disease factors suggest that psychological and personality variables may be important factors in determining vulnerability to body image distress in SSc.

There are interventions that may be useful to reduce distress related to this body image concern from SSc. For instance, cognitive-behavioral therapy for social anxiety [5] and social skills training programs [33], [34] have been recommended as strategies to reduce social avoidance and increase self-esteem in social settings for other patient groups. Rheumatologists and other healthcare professionals may consider providing patients with informational resources as a starting point in providing support and in creating an atmosphere where body image concerns and issues can be discussed among SSc patients and healthcare providers. Changing Faces, a UK not-for-profit organization (www.changingfaces.org.uk), for instance, provides workshops, as well as a range of self-help informational resources related to body image concerns from disfigurement.

There are some limitations to consider when interpreting results from the current study. The sample consisted of a convenience sample of SSc patients enrolled in the CSRG Registry; therefore, the results may not represent the full spectrum of the SSc patient population. The present sample of patients with SSc generally had stable disease (mean disease duration 10.4 years). Patients who are not cared for by a rheumatologist and patients with very severe SSc who were too sick to participate or who died earlier in their disease course were not included in the present study. This may have resulted in an over-representation of healthier patients (survival cohort), and the results may not be generalizable to the full spectrum of SSc. Related to this, statistics on the number of patients approached versus consented are not available for all CSRG centres, but it is estimated that more than 80% of patients approached to participate in the Registry do enroll. This study also used cross-sectional data that did not allow for assessment over time. Additionally, apart from the mRss, the measures of disfiguring aspects of SSc were rough and not specifically validated. For instance, manifestations of telangiectasias can be quite extensive and might contribute significantly to body image issues; however, in the current sample, measurement of telangiectasias was based on large body regions (i.e. scores across upper body parts), scored as either present or absent, and telangiectasias to specific body regions (e.g., hands or arms) could not be assessed. Similarly, the method of measuring skin pigmentation changes was limited. Hyper- and/or hypo-pigmentation was scored as either present, absent, or unknown, which is a general scoring method, and may have limited the relationship between pigmentation changes and body image distress in our models. Additionally, the high proportion of White patients in our sample may not reflect the body image changes that can occur with pigment changes in other patients, and the high proportion of White patients in the sample, relative to other ethnicities, made it impossible to examine body image changes in other groups.

In sum, this study found that both Dissatisfaction with Appearance and Social Discomfort were associated with numerous disfiguring aspects of SSc. Furthermore, younger patients were more prone to discomfort in social settings. The results of this study underline the need to attend to both disease and social contexts in understanding the impact of disfigurement on body image distress among patients with SSc and to be aware that multiple factors may contribute to patient concerns about their appearance.
